# Dapagliflozin improves diabetic cardiomyopathy by suppressing the STAT3-YY1 signaling axis in cardiac fibroblasts

**DOI:** 10.22038/ijbms.2025.87173.18843

**Published:** 2025

**Authors:** Xing-Yi Shen, Xi-Ya Li, Zuo-Ying Hu, Hao Xie

**Affiliations:** 1 Department of Cardiology, the Affiliated Suzhou Hospital of Nanjing Medical University, Suzhou Municipal Hospital, Nanjing Medical University, Suzhou, China; 2 Department of Cardiology, Nanjing First Hospital, Nanjing Medical University, Nanjing, China

**Keywords:** Dapagliflozin, Diabetic cardiomyopathy, Fibroblast

## Abstract

**Objective(s):**

Cardiac fibroblast (CF) proliferation and activation drive cardiac fibrosis and heart failure. Dapagliflozin (DAPA), a sodium-glucose cotransporter 2 (SGLT2) inhibitor, ameliorates diabetic cardiomyopathy (DCM). We investigated whether DAPA exerts anti-fibrotic and cardioprotective effects on DCM by directly suppressing CF proliferation and activation independent of SGLT2 inhibition.

**Materials and Methods:**

CFs were isolated from mouse hearts. Mouse cardiac function and fibrosis were investigated using histological analysis, western blotting, and echocardiography. Additionally, genetic loss-of-function studies were conducted *in vitro* by small interfering RNA silencing and *in vivo* by lentivirus-mediated gene knockdown.

**Results:**

Compared with high-glucose-treated neonatal rat CFs, genetic loss-of-function of signal transducer and activator of transcription 3 (STAT3) or pretreatment with DAPA dramatically inhibited STAT3 phosphorylation and Yin Yang 1 (YY1) nuclear translocation, alleviated CF proliferation and activation, and reduced fibrosis. In diabetic db/db mice, administration of DAPA remarkably ameliorated diabetes-induced STAT3 activation, YY1 nuclear translocation, CF proliferation and activation, and reduced cardiac fibrosis and dysfunction. These *in vitro* and *in vivo* effects of DAPA were ameliorated by colivelin TFA, a potent activator of STAT3. Intriguingly, knockdown of SGLT2 did not have an inhibitory effect on CF proliferation and activation in db/db mice.

**Conclusion:**

DAPA reduces cardiac fibrosis and DCM. This may, at least in part, be attributable to the repression of the STAT3-YY1 signaling axis-mediated CF proliferation and activation, independent of SGLT2 inhibition.

## Introduction

Cardiac fibrosis is a common pathophysiological companion of most myocardial diseases, including in various etiologies that induce heart failure (HF) (1, 2). As the predominant cell type in the heart, cardiac fibroblasts (CFs) proliferate and become activated, driving cardiac fibrosis and eventually HF (3, 4). Cardiac fibrosis is a primary pathological process in diabetes, and the prevalence of diabetic cardiomyopathy (DCM) is increasing in parallel with the increase in diabetes (5). DCM initially manifests as myocardial fibrosis with diastolic dysfunction and subsequent systolic dysfunction, eventually leading to clinical HF (6). Therefore, inhibiting CF proliferation and activation may be a feasible approach for the treatment of DCM. 

Yin Yang 1 (YY1) is a transcriptional repressor or activator that plays critical roles in normal processes and pathological conditions (7, 8). YY1 nuclear translocation promotes diabetic nephropathy-induced renal fibrosis (9), and YY1 is involved in cancer and pulmonary fibrosis (10, 11). Given the involvement of fibrosis in DCM (12), YY1 nuclear translocation may trigger cardiac fibrosis, leading to subsequent DCM. However, the effect of YY1 on DCM remains obscure. Signal transducer and activator of transcription 3 (STAT3) suppresses anti-fibrotic genes (13). STAT3 also mediates CF proliferation and collagen synthesis during hyperglycemia-promoted fibrosis (14). Intriguingly, YY1 interacts with the STAT3 signaling pathway (15–17). We therefore hypothesized that a YY1-STAT3 or STAT3-YY1 signaling axis may influence the initiation of cardiac fibrosis in DCM, and that identifying effective agents that inhibit this signaling axis may be beneficial for treating DCM.

Dapagliflozin (DAPA), a sodium-glucose cotransporter 2 (SGLT2) inhibitor, reduces collagen deposition and improves cardiac dysfunction in DCM (18). DAPA also attenuates cardiac fibrosis in infarcted rat hearts (19). However, whether DAPA exerts anti-fibrotic and cardio-protective effects on DCM by directly inhibiting CF proliferation and activation is not known. In this study, we demonstrate that DAPA can directly inhibit STAT3, resulting in reduced YY1 nuclear translocation, CF proliferation and activation, and cardiac fibrosis. These effects reduced cardiac dysfunction in DCM independent of SGLT2 inhibition. DAPA may therefore prevent or ameliorate DCM by suppressing the STAT3-YY1 signaling axis in CFs.

## Materials and Methods

### Regents

Dapagliflozin (#HY-10450) and colivelin TFA (#HY-P1061A) were purchased from MCE (Monmouth Junction, NJ, USA). D-Glucose (#G7528) and D-Mannitol (#M4125) were obtained from Sigma-Aldrich (St. Louis, MO, USA). Antibodies against JAK2, p-JAK2(Tyr1007/1008), STAT3, p-STAT3(Tyr705), SMAD2/3, p-SMAD2, p-SMAD3, YY1, JNK, p-JNK, p38MAPK, p-p38MAPK, PCNA, vimentin, α-SMA, histone H3, and β-actin were sourced from Cell Signaling Technology (Beverly, MA, USA), while antibodies against TGFβ receptor I, TGFβ receptor II, Collagen I, and Collagen III were purchased from Santa Cruz Biotechnology (Santa Cruz, CA, USA). An MTT Cell Proliferation and Cytotoxicity Assay Kit was purchased from Beyotime (Haimen, Jiangsu, China).

### Extraction and cultivation of neonatal rat cardiac fibroblasts (NRCFs)

NRCFs were extracted and cultured as previously described (20). Cells from passages 2–3 were used in the experiments. 

### Cell proliferation assay

NRCF proliferation was evaluated using an MTT Assay Kit according to the manufacturer’s instructions (20).

### Small interfering RNA (siRNA) transfection

Transfection of NRCFs with YY1 siRNA (ON-TARGETplus SMARTpool, L-091624-02-0005, Dharmacon Inc., Lafayette, CO, USA), STAT3 siRNA (siB150715103700-1-5, RiboBio Co., Ltd., Guangzhou, China), or Control siRNA (siN0000001-1-5, RiboBio Co., Ltd.) was performed using Lipofectamine RNAiMAX (Invitrogen, Carlsbad, CA, USA) as previously described (20, 21). All siRNA transfection experiments were performed with siRNA final concentrations of 100 nM. 

### Immunofluorescence assay

Immunofluorescence assays of NRCFs were performed as previously described (20, 21). Immunostained cells were visualized using confocal laser microscopy and analyzed using an image analysis system, as previously described (21).

### Animal studies

The onset of cardiomyopathy is more rapid and severe in diabetic females than in diabetic males (22, 23); therefore, we selected female mice as experimental subjects. Eight-week-old female C57BL/6J (18–20 g) and db/db (38–40 g) mice were obtained from Shanghai SLAC Laboratory Animal Co., Ltd (Shanghai, China). Animals were housed under standard conditions with a 12-hr light/dark cycle and access to distilled water and chow *ad libitum*. Mice were randomly divided into eight groups: C57BL/6J mice (Control, n=6); C57BL/6J mice intragastrically administered 1.5 mg/kg DAPA (AstraZeneca Co, Mount Vernon, IN, USA) once per day (Control+DAPA, n=6); C57BL/6J mice intraperitoneally injected with 1 mg/kg colivelin TFA (MedChem Express) once per day (Control+TFA, n=6); C57BL/6J mice intraperitoneally injected with 1 mg/kg colivelin TFA once per day for 2 weeks and then with the same dose of colivelin TFA and 1.5 mg/kg DAPA once per day (Control+TFA+DAPA, n=6); *db*/*db* mice (DM, n=8); *db*/*db* mice intragastrically administered 1.5 mg/kg DAPA once per day (DM+DAPA, n=8); *db*/*db* mice intraperitoneally injected with 1 mg/kg colivelin TFA once per day (DM+TFA, n=8);* db*/*db* mice intraperitoneally injected with 1 mg/kg colivelin TFA once per day for 2 weeks and then with the same dose of colivelin TFA and 1.5 mg/kg DAPA once per day (DM+TFA+DAPA, n=8). Mice were intragastrically administered DAPA for 8 weeks for subsequent analyses as previously described (18).

### Lentivirus injection

To elucidate whether SGLT2 inhibition exerts an anti-fibrotic effect in diabetic mice, the *Sglt2* gene was knocked down using small hairpin RNA (shRNA) delivered using a lentivirus (LV). LV-SGLT2 shRNA (titer: 3.0×10^9^ TU/ml) and LV-Scrambled shRNA (titer: 6.3 × 10^9^ TU/ml) were generated by Genechem (Shanghai, China). The SGLT2 and Scrambled shRNAs sequences were GCCTTCATCCTCACTGGTTAT and TTCTCCGAACGTGTCACGT, respectively. Eight-week-old female *db*/*db* mice (n=30) were arbitrarily allocated into three groups: *db*/*db* treated with normal saline, *db*/*db *treated with LV-Scrambled shRNA, and *db*/*db *treated with LV-SGLT2 shRNA. To test the efficiency of the SGLT2 knockdown, age-matched female C57BL/6J mice (n=18) were divided into three groups: control, control treated with LV-Scrambled shRNA, and control treated with LV-SGLT2 shRNA. Using a 1 ml insulin syringe, 20 μl of LV-SGLT2, or LV-Scrambled shRNA was slowly injected into the tail vein. After 8 weeks, cardiac function was assessed, and other experiments were performed.

### Echocardiography

All echocardiography testing indexes were examined as previously described (20, 24).

### Adult mouse left ventricular cardiac fibroblast (AMCF) preparation

To determine whether SGLT2 inhibition exerts anti-fibrotic and cardio-protective effects on DCM by directly inhibiting CF proliferation and activation, left ventricular AMCFs were extracted and cultured as previously described (25, 26). Briefly, adult female mice were anaesthetized with 1% pentobarbital sodium (50 mg/kg intraperitoneal injection) and euthanized by cervical dislocation. AMCFs were then prepared using the protocol for preparing NRCFs. 

### Preparation of cytoplasmic and nuclear proteins

Cytoplasmic and nuclear proteins from NRCFs and AMCFs were prepared following our previously published methods (25) using NE-PER Nuclear and Cytoplasmic Extraction Reagents (Pierce Biotechnology, Inc., Rockford, IL, USA). 

### Western blotting and immunoprecipitation

Proteins were extracted from cells for western blotting and immunoprecipitation analyses following previously described methods (21).

### Histology

Mouse hearts were isolated and fixed in 4% paraformaldehyde solution, and embedded in paraffin. Five-micron-thick sections of the left ventricle were prepared and stained with Massonʼs trichrome for evaluation of fibrosis content according to previously described methods (20, 25, 26). To investigate CF activation *in vivo*, the accumulation of Collagen I and Collagen III was determined using immunohistochemistry according to previously published methods (20, 25, 26). In addition, SGLT2 knockdown efficiency was verified in the kidney using immunohistochemistry. Stained and immunostained sections were visualized and analyzed according to our previously published methods (21, 25).

### Statistical analysis

Data are presented as the mean ± standard error of the mean (SEM). Differences in data between groups were compared with Student’s t-test or one-way ANOVA analysis. When statistical significance was found with ANOVA, a *post hoc* Tukey test for multiple comparisons was performed. Statistical analyses were performed and graphs were generated using GraphPad PRISM software 8.0 (GraphPad Inc., San Diego, CA, USA). A *P*-value≤0.05 was considered statistically significant.

## Results

### High-glucose (HG) activates canonical and non-canonical TGFβ pathways in vitro

To test whether DAPA has an inhibitory effect on canonical and/or non-canonical TGFβ pathways in HG-treated NRCFs, we performed numerous western blot assays. As shown in Supplementary Figure 1A and 1B, phosphorylated SMAD2, SMAD3, and TAK1, and increased levels of TβRI and TβRII were observed in HG-cultured NRCFs, whereas total levels of SMAD2, SMAD3, and TAK1 were not changed. These data show that canonical and non-canonical TGFβ pathways were activated in HG-treated NRCFs.

### YY1’s role in HG-induced NRCF proliferation and activation, as well as in the up-regulation of extracellular matrix (ECM) protein levels.

**Figure 1:**
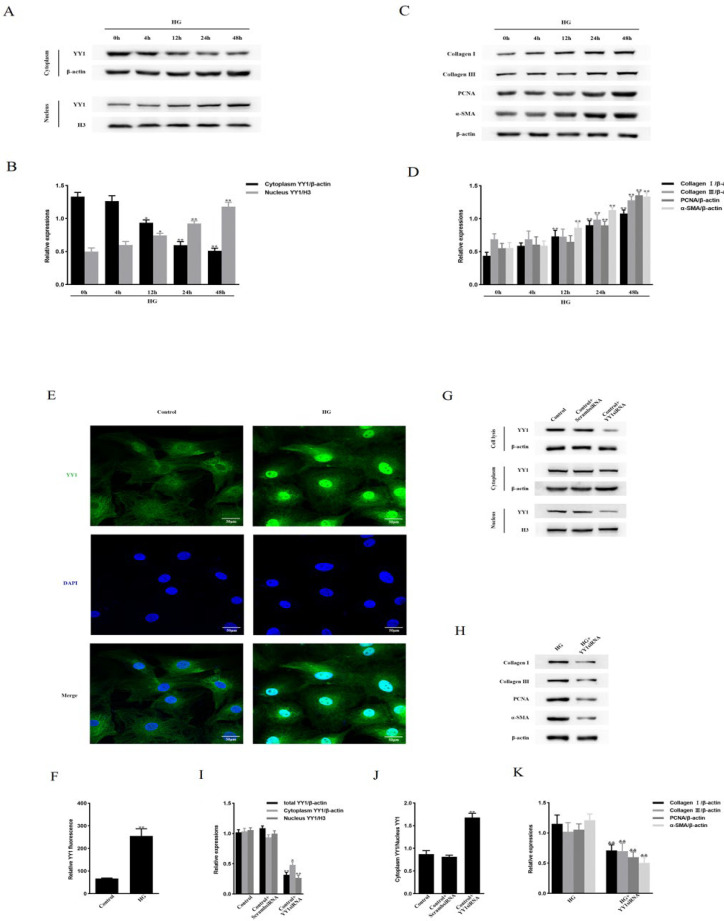
YY1 mediates NRCF proliferation and activation, and up-regulation of ECM proteins under hyperglycemic conditions

NRCFs were stimulated with HG (25 mmol/l) for different periods. YY1 nuclear translocation was enhanced (Figure 1A, 1B), and the levels of Collagen I, Collagen III, PCNA, and α-SMA (Figure 1C, 1D) were elevated at 12 hr and peaked at 48 hr. YY1 nuclear translocation was also confirmed in HG-induced NRCFs by immunofluorescence (Figure 1E, 1F). Next, to verify whether the proliferation and activation of HG-induced NRCFs were mediated by YY1, we transfected NRCFs with or without YY1 siRNA. As shown in Figure 1G, in contrast to control transfected cells, YY1 siRNA reduced YY1 protein levels by approximately 68.8%. The level of YY1 in the nucleus was lowered by 74.5%, and in the cytoplasm by 45.1%; the YY1 cytoplasm/nucleus ratio was 1.68 (Figure 1G, 1I, 1J), which indicated that the net effect of YY1 siRNA was to prevent YY1 nuclear translocation. In addition, NRCFs treated with YY1 siRNA and then stimulated with HG for 48 hr had markedly decreased levels of Collagen I, Collagen III, PCNA, and α-SMA (Figure 1H, 1K). We then examined the effects of HG on NRCF proliferation. Cell proliferation was observed after 12 hr of HG-stimulation and reached its highest level at 48 hr (Supplementary Figure 2A). The proliferative effect of HG on NRCFs was attenuated by transfection of YY1 siRNA (Supplementary Figure 2B). However, treatment with YY1 siRNA did not affect levels of p-TAK1, TβRI, TβRII, p-SMAD2, or p-SMAD3 in HG-cultured NRCFs (Supplementary Figure 2C, 2D). These findings indicate that YY1 participates in HG-stimulated NRCF proliferation and activation and that YY1 up-regulates ECM proteins, independent of TGFβ signaling inhibition. 

### STAT3 engages in YY1-mediated cell proliferation and activation and in the up-regulation of ECM protein levels in HG-treated NRCFs

**Figure 2:**
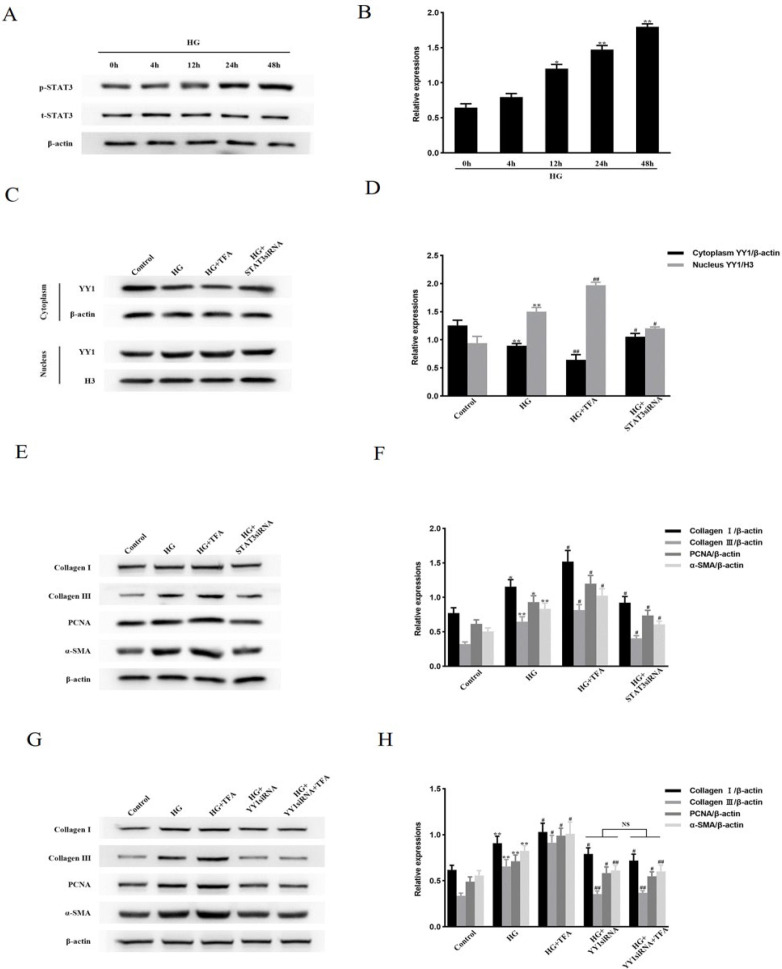
STAT3 is involved in YY1-mediated NRCF proliferation and activation and in the up-regulation of ECM protein levels under hyperglycemia conditions

NRCFs were stimulated with HG for different periods. The phosphorylation of STAT3 at Tyr705 in HG-cultured NRCFs was augmented at 12 hr and reached its highest level at 48 hr, while the total level of STAT3 did not change (Figure 2A, 2B). Next, the interaction between YY1 and STAT3 was determined in HG-treated NRCFs. NRCFs were treated with YY1 siRNA and then stimulated with HG. Neither the total nor the phosphorylated level of STAT3 changed (Supplementary Figures 3A and 3B). This indicated that STAT3 was not downstream of YY1. In addition, we verified the efficiency of the STAT3 siRNA knockdown. STAT3 siRNA lowered STAT3 levels by approximately 67.5%, while the control siRNA had no effect (Supplementary Figure 3C, 3D). Pretreatment of NRCFs with colivelin TFA (a potent activator of STAT3) followed by stimulation with HG prominently enhanced YY1 nuclear translocation and increased the levels of Collagen I, Collagen III, PCNA, and α-SMA. By contrast, these effects were markedly reduced by transfection of STAT3 siRNA (Figure 2C–F). However, neither TFA nor STAT3 siRNA treatment affected the levels of p-TAK1, TβRI, TβRII, p-SMAD2, or p-SMAD3 in HG-cultured NRCFs (Supplementary Figure 3E, 3F). Notably, treatment with YY1 siRNA followed by TFA administration significantly attenuated the up-regulation of ECM protein levels in HG-stimulated NRCFs (Figure 2G, 2H). These findings demonstrate that STAT3 participates in YY1-mediated cell proliferation and activation, as well as in the up-regulation of ECM protein levels in HG-stimulated NRCFs.

### SGLT2 knockdown does not inhibit YY1 nuclear translocation, CF proliferation and activation, or up-regulation of ECM protein level in db/db mice

**Figure 3:**
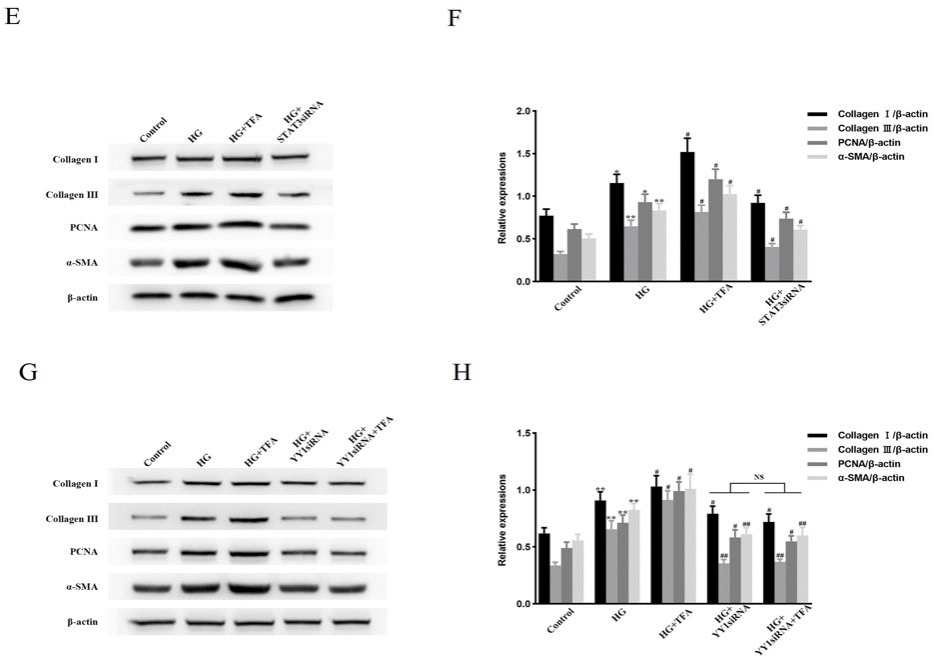
Effect of SGLT2 knockdown on YY1 nuclear translocation, CF proliferation and activation, and ECM protein up-regulation in db/db mice

To investigate whether the inhibition of YY1 nuclear translocation and CF proliferation and activation, and the reduction in ECM proteins levels by DAPA is dependent on the suppression of SGLT2, we isolated CFs from db/db mice with or without SGLT2 knockdown. First, the efficiency of SGLT2 shRNA knockdown in the kidney was evaluated by immunohistochemistry. As shown in Supplementary Figure 4A and 4B, compared with wild-type (WT) mice, treatment with SGLT2 shRNA lowered SGLT2 levels in the kidney by approximately 84.2%. Similar results were observed in db/db mice treated with or without SGLT2 shRNA; SGLT2 levels were reduced by approximately 67.5% (Figures 3A and 3B). However, SGLT2 knockdown had no effect on YY1 nuclear translocation, CF proliferation and activation, or ECM protein levels in CFs from db/db mice (Figure 3C, 3D, 3E, 3F). These findings show that DAPA inhibits YY1 nuclear translocation, CF proliferation and activation, and reduces ECM protein levels independent of SGLT2 inhibition. 

### DAPA does not inhibit TGFβ signaling in either HG-treated NRCFs or diabetes-stimulated CFs

We determined whether DAPA inhibits canonical and/or non-canonical TGF-β signaling in HG-treated NRCFs and diabetes-stimulated CFs. Phosphorylated SMAD2, SMAD3, and TAK1, and increased levels of TβRI and TβRII were observed in HG-cultured NRCFs and diabetes-stimulated CFs, whereas total SMAD2, SMAD3, and TAK1 were not changed. These HG or diabetes-induced effects were not inhibited by DAPA (Supplementary Figure 4C–F). These data show that DAPA does not inhibit canonical or non-canonical TGF-β signaling in HG-treated NRCFs and diabetes-stimulated CFs. 

### DAPA inhibits STAT3 suppression-dependent YY1 nuclear translocation, cell proliferation, activation, and ECM protein level up-regulation in HG-treated NRCFs

**Figure 4:**
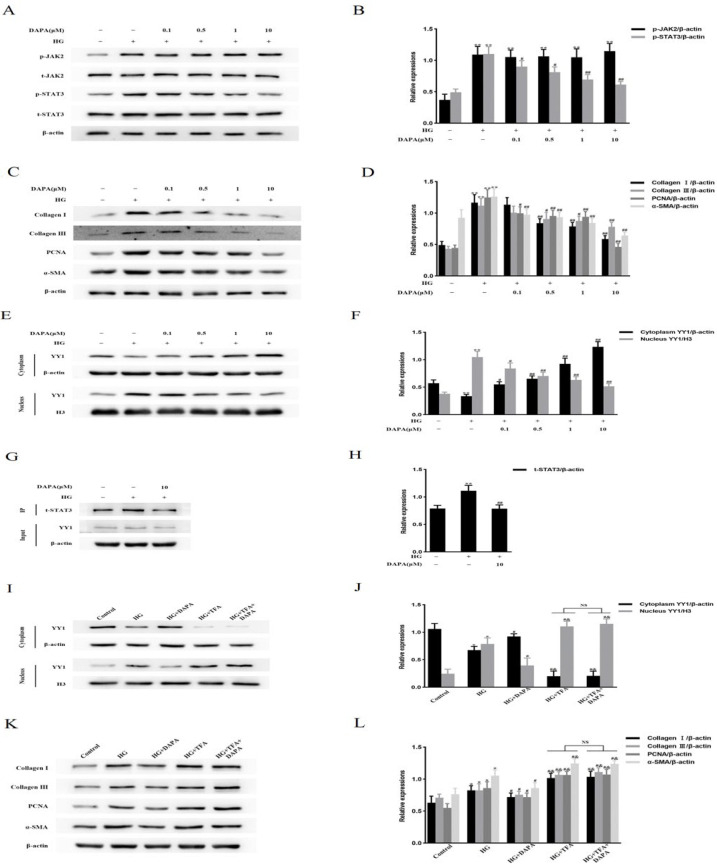
DAPA inhibition of YY1 nuclear translocation and fibrogenic protein levels is dependent on STAT3 suppression in NRCFs under hyperglycemic conditions

Phosphorylated STAT3 and the levels of Collagen I, Collagen III, PCNA, and α-SMA were substantially decreased in HG-treated NRCFs pretreated with DAPA, while phosphorylated JAK2 (upstream of STAT3) and total levels of STAT3 and JAK2 were unchanged (Figure 4A–D). YY1 nuclear translocation was also markedly suppressed by treatment of HG-cultured NRCFs with DAPA (Figure 4E, 4F). Next, the interaction between YY1 and STAT3 under HG stimulation with or without DAPA was determined by immunoprecipitation. The binding of STAT3 to YY1 was enhanced by HG stimulation, which was suppressed by DAPA (Figure 4G, 4H). These findings suggest that DAPA may inhibit YY1 in HG-treated NRCFs by disrupting STAT3-YY1 complexes. To verify whether DAPA inhibition of YY1 nuclear translocation and cell proliferation and activation in HG-treated NRCFs was dependent on STAT3 suppression, cells were pretreated for 1 hr with or without colivelin TFA and then incubated with or without DAPA for another 1 hr before HG stimulation for the indicated periods. Compared with NRCFs that were only treated with or without DAPA before exposure to HG, the amount of YY1 nuclear translocation and the levels of Collagen I, Collagen III, PCNA, and α-SMA were increased in cells pretreated with colivelin TFA and incubated with or without DAPA after exposure to HG (Figure 4I, 4J, 4K, 4L). These data indicate that DAPA inhibition of HG-induced NRCF proliferation and activation was dependent on inactivation of the STAT3-YY1 signaling axis.

### DAPA attenuates diabetes-induced and STAT3-dependent YY1 nuclear translocation, CF proliferation and activation, and up-regulation of ECM protein levels

**Figure 5:**
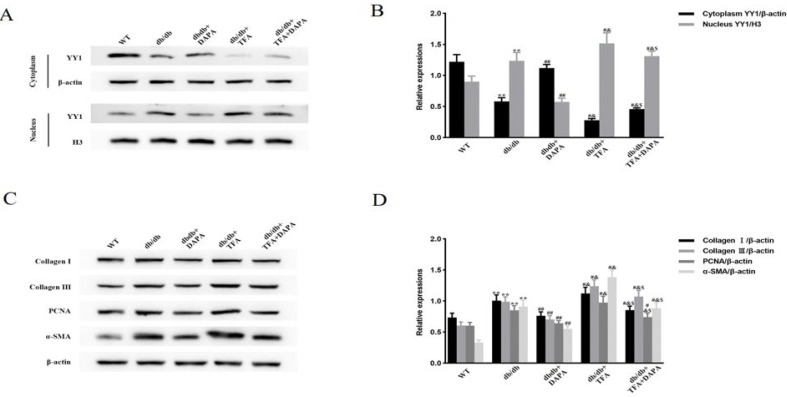
Effects of DAPA on YY1 nuclear translocation, CF proliferation and activation, and ECM protein up-regulation in db/db mice are dependent on STAT3

In contrast to CFs from db/db mice, YY1 nuclear translocation and the levels of Collagen I, Collagen III, PCNA, and α-SMA were significantly enhanced in CFs from db/db mice after intraperitoneal injection of colivelin TFA. These increases were substantially suppressed by the addition of DAPA (Figures 5A and 5B). Intriguingly, treatment with colivelin TFA followed by administration of DAPA partly attenuated the inhibitory effect of DAPA on CFs from db/db mice (Figure 5C, 5D). These data showed that the DAPA inhibition of YY1 nuclear translocation, CF proliferation and activation, and up-regulation of ECM proteins in db/db mice was, at least in part, dependent on STAT3.

### SGLT2 knockdown reduces cardiac fibrosis and cardiac dysfunction in db/db mice

**Figure 6:**
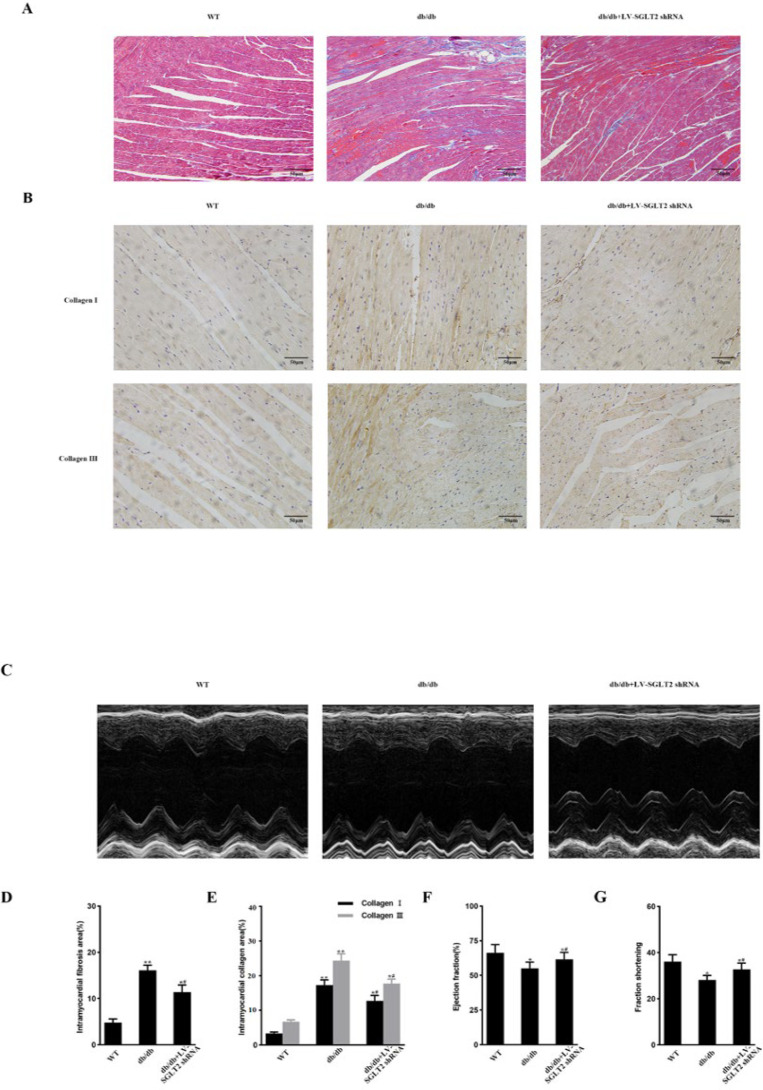
Cardiac fibrosis and dysfunction are ameliorated via SGLT2 knockdown

SGLT2 shRNA treatment substantially mitigated diabetes-induced cardiac fibrosis in db/db mice, as shown by Massonʼs staining and immunohistochemistry (Figure 6A, 6B, 6D, and 6E). Additionally, systolic dysfunction in db/db mice was ameliorated by administration of LV-SGLT2 shRNA (Figure 6C, 6F, and 6G). Cardiac fibrosis and cardiac function did not change in WT mice treated with SGLT2 shRNA compared with untreated WT mice (data not shown). These data indicate that SGLT2 knockdown exerted a cardioprotective effect on db/db mice.

### DAPA attenuation of diabetic cardiomyopathy is partly dependent on STAT3

**Figure 7:**
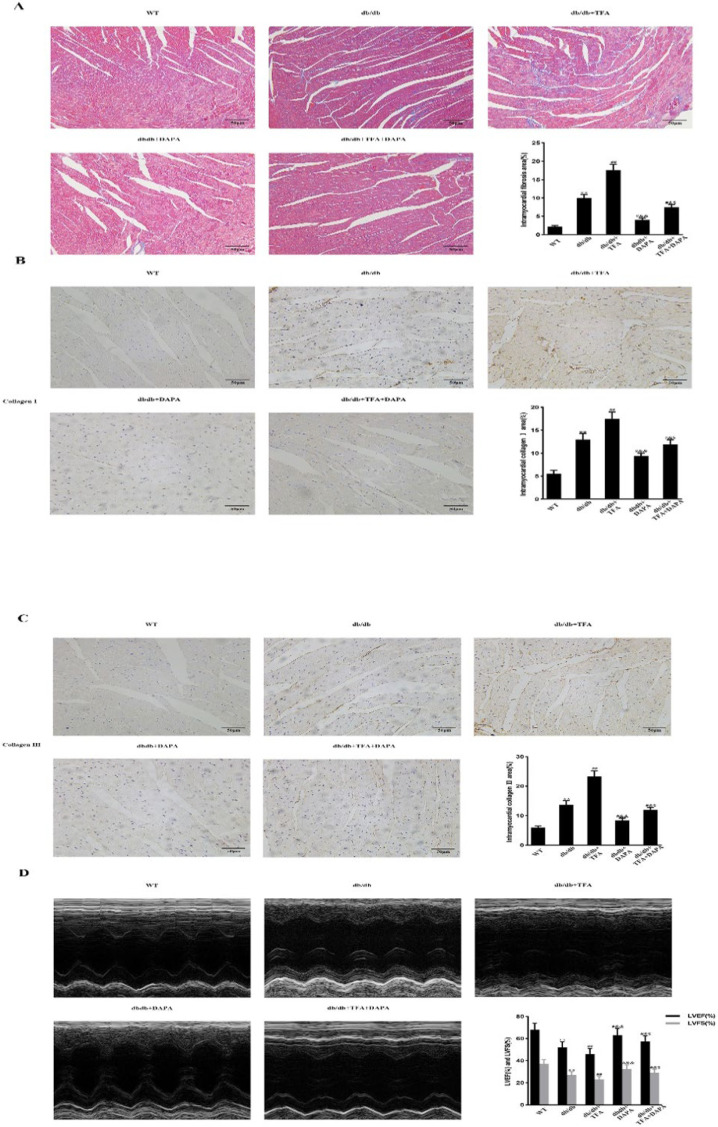
DAPA attenuation of myocardial fibrosis and cardiac dysfunction is partly dependent on STAT3

Cardiac fibrosis and dysfunction were markedly reduced in db/db mice treated with DAPA compared with untreated db/db mice. These effects were ameliorated by treatment with colivelin TFA. It should be noted that the impact of colivelin TFA on db/db mice were partly attenuated by the administration of DAPA (Figure 7A–7D). Cardiac fibrosis and dysfunction in WT mice treated with DAPA or colivelin TFA were not different from those in untreated WT mice (data not shown). These data indicated that DAPA alleviation of myocardial fibrosis and dysfunction in db/db mice was partly dependent on STAT3 inhibition. 

## Discussion

DCM is defined as ventricular diastolic and/or systolic dysfunction in diabetic individuals that cannot be ascribed to coronary disease, hypertension, or other heart diseases (6, 27) and that cannot be effectively reversed by intensive blood glucose control (28). Therefore, understanding the underlying mechanism of DCM is critical to improving its treatment. The pathophysiology of DCM is multifactorial, with cardiac fibrosis being a major factor (28). A mouse model of DCM shows gradually increasing fibrosis and remarkable deterioration of cardiac function. Anti-fibrotic treatment effectively improved DCM in this model (20), but specific pharmaceuticals directly targeting fibrosis are still lacking (5). DAPA impedes cardiac fibrosis and improves cardiac dysfunction in DCM (18). As the predominant cell type in the heart, CFs proliferate and become activated in DCM, driving ECM remodeling and cardiac fibrosis, eventually leading to HF (3, 4). Here, we investigated whether DAPA exerts anti-fibrotic and cardioprotective effects on DCM by directly inhibiting CF proliferation and activation.

SGLT2 inhibition reduces HF, which may be related to natriuresis, osmotic diuresis, and plasma volume contraction (6). Moreover, Satou *et al.* reported that blockade of SGLT2 suppresses HG-induced angiotensinogen augmentation in renal proximal tubular cells (29). Angiotensinogen is a precursor of angiotensin I (Ang I) and Ang II, which promote CF proliferation and activation in HF (30); therefore, improvement of cardiac dysfunction by SGLT2 shRNA treatment is consistent with this effect. However, administration of DAPA, but not knockdown of SGLT2, markedly inhibited cell proliferation and activation, and reduced ECM protein levels in CFs isolated from hearts of db/db mice. SGLT2 is not expressed in the heart (6, 31); therefore, these DAPA effects on CFs must be independent of SGLT2 inhibition. Next, we determined whether DAPA exerted an inhibitory effect on canonical and/or non-canonical TGFβ signaling, which mediates CF activation and the progression of fibrogenesis (32, 33). Proteins involved in signal transduction, including TβRI, TβRII, SMAD2, SMAD3, and TAK1, were unexpectedly up-regulated or activated* in vitro* and *in vivo*, but were not suppressed by DAPA. These findings suggest that the effects of DAPA on CF proliferation and activation may be independent of its inhibition of TGFβ signaling. Therefore, identifying the molecular target of DAPA that inhibits CF proliferation and activation is of great significance for the treatment of DCM.

YY1 is a transcriptional repressor or activator that plays a vital role in cell proliferation (7, 8). YY1 can promote fibrosis under certain conditions, such as diabetic nephropathy and pulmonary fibrosis (9, 10). We hypothesized that YY1 mediates diabetes-associated cardiac fibrosis. Indeed, YY1 participated in hyperglycemia-induced CF proliferation and activation, ECM protein up-regulation, and subsequent myocardial fibrosis. Interestingly, these effects were reversed by DAPA, indicating that YY1 is an effective molecular target of DAPA in the treatment of DCM. However, whether DAPA suppresses YY1 directly or via upstream molecules is unknown and requires further research.

STAT3 mediates the proliferation of CFs and subsequent collagen synthesis and is involved in hyperglycemia-promoted fibrosis (14). Accumulating evidence shows that YY1 may cooperate with the STAT3 signaling pathway (15–17). We therefore suspected that a YY1-STAT3 or STAT3-YY1 signaling axis would engage in DAPA anti-fibrotic and cardioprotective effects. We found that HG or diabetes enhanced STAT3 binding to YY1, which DAPA suppressed. Therefore, DAPA may exert an inhibitory effect on YY1 by disrupting STAT3-YY1 complexes. Pretreatment of CFs with colivelin TFA markedly enhanced hyperglycemia-induced YY1 nuclear translocation, cell proliferation and activation, and ECM protein up-regulation, while DAPA did not reverse these effects. These findings show that DAPA inhibition of YY1 is dependent on STAT3 inhibition. Colivelin TFA treatment increased cardiac fibrosis and cardiac insufficiency in diabetic mice. However, DAPA only partially alleviated this effect. This may be because STAT3 is also expressed in non-cardiac fibroblasts, and these cells may promote cardiac remodeling *in vivo* (19). Furthermore, a body of evidence suggests that leptin-STAT3 signaling in the central nervous system is involved in the development of obesity and diabetes (34–37). Colivelin TFA is a neuroprotective peptide that can cross the blood–brain barrier (38) and, therefore, interact with leptin-STAT3 signaling in the central nervous system to promote diabetes and DCM.

(A, C) NRCFs were stimulated with HG for different periods. The extent of YY1 nuclear translocation and the levels of Collagen I, Collagen III, PCNA, and α-SMA were examined by western blotting. (B, D) The column figures were drawn for (A) and (C), respectively. (E) NRCFs were stimulated with HG for 48 hr. The extent of YY1 nuclear translocation was evaluated by immunofluorescence. (G) Total and nuclear amounts of YY1 in NRCFs treated with or without YY1 siRNA or Scrambled siRNA were examined by western blotting. (H) Levels of Collagen I, Collagen III, PCNA, and α-SMA were evaluated by western blotting in NRCFs treated with or without YY1 siRNA and then stimulated with HG for 48 hr. (F, I, J, K) The column figures were depicted for (E, G, H), respectively. Data in (A, C, E, G, H) were from three independent experiments. *P<0.05, **P<0.01 vs Control; &&*P*<0.01 vs HG. HG: High-glucose; YY1: Yin Yang 1

(A, B) NRCFs were stimulated with HG for different periods. Total and phosphorylated levels of STAT3 were examined by western blotting. (C, D, E, F) NRCFs were pretreated with colivelin TFA at 50 µg/ml for 1 hr or transfected with STAT3 siRNA for 24 hr and then stimulated with HG for 48 hr. The amount of YY1 nuclear translocation (C, D) and the levels of Collagen I, Collagen III, PCNA, and α-SMA (E, F) were then examined by western blotting. (G, H) NRCFs were treated with or without YY1 siRNA, and then incubated with or without colivelin TFA at 50 µg/ml, and then stimulated with HG for 48 hr. Collagen I, Collagen III, PCNA, and α-SMA levels were then evaluated by western blotting. All data were from three independent experiments. *P<0.05, **P<0.01 vs Control; #*P*<0.05, ##*P*<0.01 vs HG; Not significant (NS), *P*>0.05. HG: High-glucose; TFA: Colivelin TFA

(A, B) db/db mice treated with or without lentivirus-packaged SGLT2 shRNA (LV-SGLT2 shRNA) were evaluated for SGLT2 levels in the kidneys by immunohistochemistry after 8 weeks. (C, D, E, F) The extent of YY1 nuclear translocation and the levels of Collagen I, Collagen III, PCNA, and α-SMA in CFs of db/db mice treated with or without LV-SGLT2 shRNA were examined by western blotting. Data in B were from six independent experiments. Data in D and F were from three independent experiments. **P<0.01 vs WT; ##*P*<0.01 vs db/db

YY1, Yin Yang 1; WT, wild-type mice; db/db + LV-Scrambled shRNA, diabetic mice treated with LV-Scrambled shRNA; db/db +LV-SGLT2 shRNA, diabetic mice treated with LV- SGLT2 shRNA

(A, B) NRCFs were incubated with or without DAPA at the indicated concentrations for 2 hr and then stimulated with HG for 48 hr. Phosphorylated JAK2 and STAT3 and total JAK2 and STAT3 were examined by western blotting. (C, D, E, F) In cells treated as described in (A), the levels of Collagen I, Collagen III, PCNA, and α-SMA, and the amount of YY1 nuclear translocation were evaluated by western blotting. (G, H) NRCFs were or were not administered DAPA (10 μM) and then stimulated with HG for 48 hr. YY1 was immunoprecipitated from cell lysates, and the level of STAT3 in the immunoprecipitates was determined by western blotting. (I, J, K, L) The amount of YY1 nuclear translocation and the levels of Collagen I, Collagen III, PCNA, and α-SMA in NRCFs treated with or without TFA combined with or without DAPA, and then stimulated with HG for another 48 hr, were examined by western blotting. All data were from three independent experiments. *P<0.05, **P<0.01 vs Control; #*P*<0.05, ##P<0.01 vs HG; &*P*<0.05 vs HG + DAPA; Not significant (NS), *P*>0.05. YY1: Yin Yang 1; HG: High-glucose; DAPA: Dapagliflozin; TFA: Colivelin TFA

(A, B, C, D) The amount of YY1 nuclear translocation and the levels of Collagen I, Collagen III, PCNA, and α-SMA in CFs isolated from the hearts of db/db mice treated with or without TFA combined with or without DAPA were examined by western blotting. Data in (A–D) were from five independent experiments. **P<0.01 vs WT; #*P*<0.05, ##*P*<0.01 vs db/db; &*P*<0.05 vs db/db + DAPA; $*P*<0.05 vs db/db + TFA

DAPA: Dapagliflozin; TFA: Colivelin TFA; WT: Wild-type mice; db/db + DAPA: db/db mice treated with DAPA; db/db + TFA: db/db mice treated with colivelin TFA; db/db + TFA + DAPA: db/db mice treated with colivelin TFA combined with DAPA

(A, B) The extent of cardiac fibrosis in WT and db/db mice treated with or without LV-SGLT2 shRNA was examined by Massonʼs staining and immunohistochemistry (original magnification ×400). Scale bars, 50 μm. (C) Representative M-mode echocardiograms are exhibited for each group of mice treated as described in (A) and (B). (D) and (E) show quantification of the differences in collagen deposition, and (F) and (G) exhibit quantification of the differences in left ventricular ejection fraction and fractional shortening. n = 6 in each group. *P<0.05, **P<0.01 vs WT; #P<0.05 vs db/db. WT: Wild-type mice; db/db + LV-SGLT2 shRNA: db/db mice treated with LV-SGLT2 shRNA

(A, B, C) The extent of cardiac fibrosis in WT and db/db mice that were or were not administered TFA combined with or without DAPA was examined by Massonʼs staining and immunohistochemistry (original magnification ×400). Scale bars, 50 μm. (D) Representative M-mode echocardiograms of mice treated as described in (A) are shown for each group. Differences in collagen deposition (A, B, C) and left ventricular ejection fraction and fraction shortening (D) were observed. n = 6 in each group. **P<0.01 vs WT; #*P*<0.05, ##*P*<0.01 vs db/db; &*P*<0.05, &&*P*<0.01 vs db/db + TFA; $P<0.05 vs db/db + DAPA

WT: Wild-type mice; db/db + DAPA: db/db mice treated with DAPA; db/db + TFA: db/db mice treated with colivelin TFA; db/db + TFA + DAPA: db/db mice treated with colivelin TFA combined with DAPA.

## Conclusion

DAPA may directly suppress the STAT3-YY1 signaling axis in CFs, leading to reduced CF proliferation and activation, decreased myocardial fibrosis, and improved cardiac insufficiency in DCM. These findings indicate that DAPA is a potential anti-fibrotic drug for the treatment of DCM and other fibrotic heart diseases (26).

## Data Availability

The raw data supporting the conclusions of this article are available from the corresponding author upon reasonable request.

## Declaration

We have not utilized any AI tools or technologies in the preparation of this manuscript.
