# Supplementary material for: Dapagliflozin improves diabetic cardiomyopathy by suppressing the STAT3-YY1 signaling axis in cardiac fibroblasts
Source: Iran J Basic Med Sci. 2025;28(11):1563–74. doi: 10.22038/ijbms.2025.87173.18843 (PMC12571176; doi:10.22038/ijbms.2025.87173.18843)
Supplement: Supplementary file 1 — Supplementary Figures 1-4 [file ijbms-28-1563-s001.pdf]

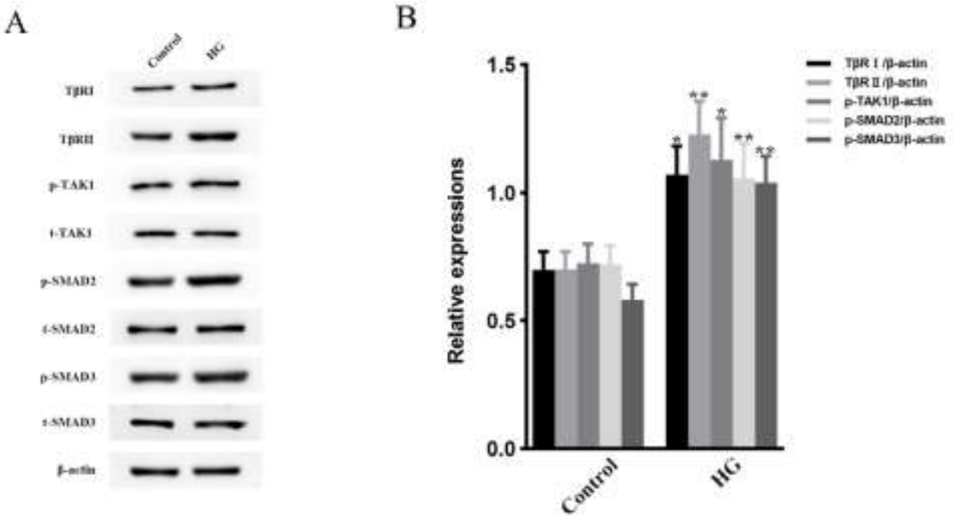

Supplementary Figure 1. HG induces activation of canonical and noncanonical TGFβ pathways in NRCFs

(A, B) NRCFs were cultured in HG medium for 48 h. Phosphorylated SMAD2, SMAD3, and TAK1, and total protein levels of TβRI, TβRII, SMAD2, SMAD3, and TAK1 were then detected by western blotting. All data were obtained from three independent experiments. *P<0.05, **P<0.01 *vs.* Control

HG: high glucose; NRCFs: neonatal rat cardiac fibroblasts

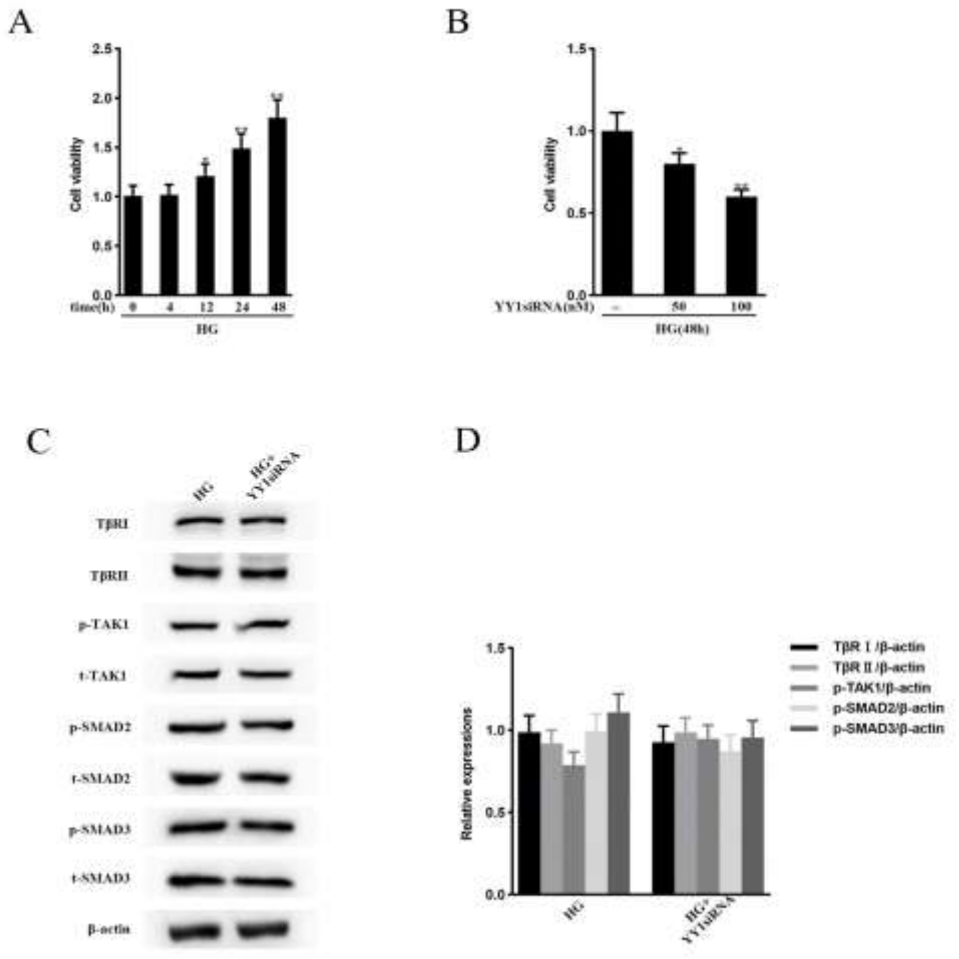

Supplementary Figure 2. The effect of YY1 knockdown on HG-induced NRCF proliferation and canonical and noncanonical TGFβ pathways

(A) NRCFs were exposed to HG for 0–48 h. Cell viability was detected by MTT assays. (B) NRCFs were transfected with or without YY1siRNA at the indicated concentrations for 24 h and then exposed to HG for 48 h. Cell viability was detected by MTT assays. (C, D) In cells treated as described in (B), phosphorylated SMAD2, SMAD3, and TAK1, and total protein levels of TβRI, TβRII, SMAD2, SMAD3, and TAK1 were detected by western blotting. All data were obtained from three independent experiments. *$P<0.05$, **$P<0.01$ *vs.*0 h, 4 h or HG

HG: high glucose

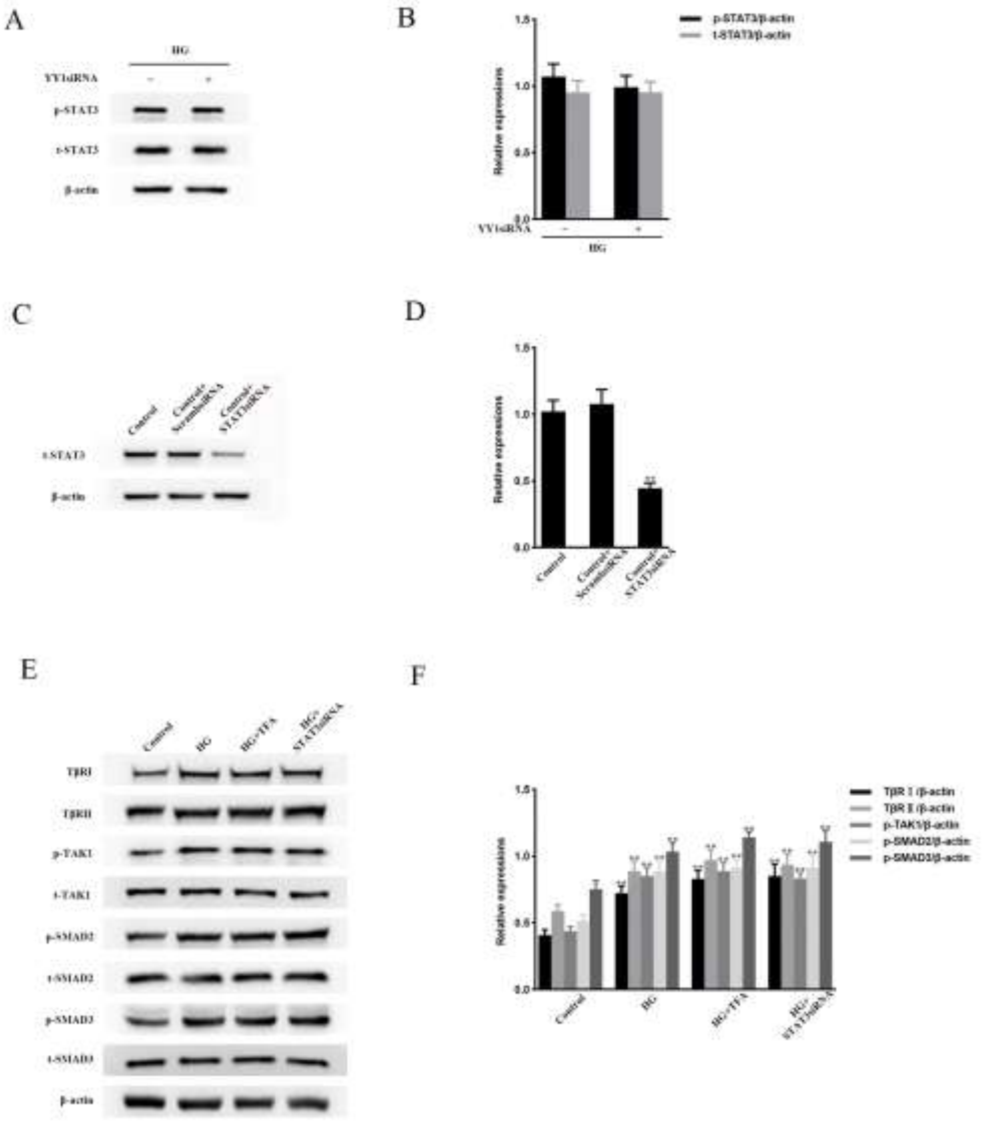

Supplementary Figure 3. The effect of STAT3 knockdown on HG-induced canonical and noncanonical TGFβ pathways in NRCFs

(A, B) NRCFs were transfected with or without YY1 siRNA for 24 h and then exposed to HG for 48 h. Phosphorylated STAT3 and total protein levels of STAT3 were detected by western blotting. (C, D) NRCFs were transfected with or without STAT3 siRNA for 48 h and then total STAT3 levels were detected by western blotting. (E, F) NRCFs were pretreated with colivelin TFA (a potent activator of STAT3) at 50 μg/ml for 1 h or transfected with STAT3 siRNA for 24 h and then exposed to HG for 48 h. Phosphorylated SMAD2, SMAD and TAK1, and total TβRI, TβRII, SMAD2, SMAD3, and TAK1 levels were detected by western blotting. All data were obtained from three independent experiments. **P<0.01 *vs.* Control

HG: high glucose; TFA: colivelin TFA

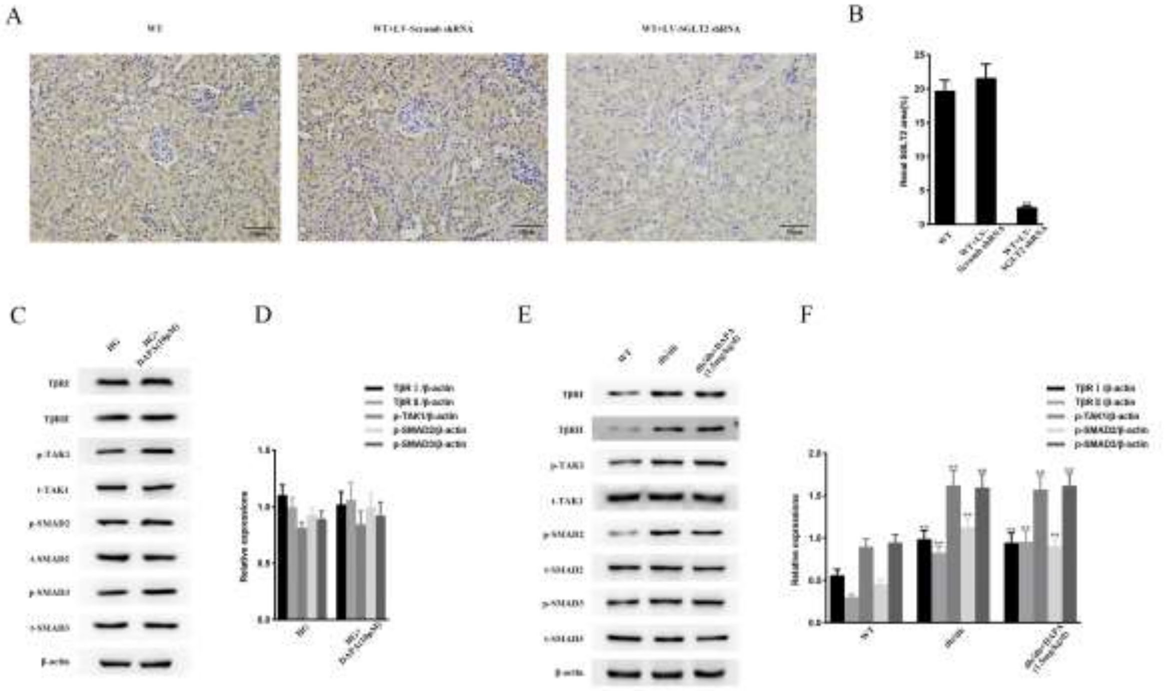

Supplementary Figure 4. The effect of SGLT2 knockdown on canonical and noncanonical TGFβ pathways in HG or diabetes-stimulated CFs

(A, B) Wild-type mice with or without tail vein injection of lentivirus-packaged SGLT2 shRNA (LV-SGLT2 shRNA) were evaluated for SGLT2 in the kidneys by immunohistochemistry after 8 weeks. (C, D) NRCFs were pretreated with dapagliflozin at the indicated concentrations for 1 h and then exposed to HG for 48 h. Phosphorylated SMAD2, SMAD3, and TAK1, and total TβRI, TβRII, SMAD2, SMAD3, and TAK1 levels were detected by western blotting. (E, F) CFs were isolated from wild-type mice or db/db mice treated with or without dapagliflozin (1.5 mg/kg/d) and then phosphorylated SMAD2, SMAD3, and TAK1, and total TβRI, TβRII, SMAD2, SMAD3, and TAK1 levels were detected by western blotting. All data were obtained from three independent experiments. ** P<0.01 *vs.* WT

WT: wild-type mice; db/db: diabetic mice; DAPA: dapagliflozin; WT + LV-Scrambled shRNA: WT mice treated with LV-Scrambled shRNA; WT + LV-SGLT2 shRNA: WT mice treated with LV-SGLT2 shRNA; db/db + DAPA: db/db mice treated with dapagliflozin
